# Interpretable self-supervised contrastive learning for colorectal cancer histopathology: GRADCAM visualization

**DOI:** 10.6026/973206300211836

**Published:** 2025-07-31

**Authors:** Tarun Jain, Andrew M. Lynn

**Affiliations:** 1 School of Computational and Integrative Sciences (SCIS), Jawaharlal Nehru University, New Delhi, India

**Keywords:** Self supervised contrastive learning, colorectal cancer histopathology, deep learning, interpretable AI, GradCAM

## Abstract

Accurate colorectal cancer diagnosis from histopathological images is crucial for effective treatment. Therefore, it is of interest to describe a novel framework that combines self-supervised contrastive learning (SSCL) with Grad-CAM-based interpretability for classifying hyperplastic polyp (HP) and sessile serrated adenoma (SSA). A ResNet50 encoder is first pre-trained using SSCL to learn rich feature representations from unlabeled images, minimizing the need for manual annotations which are then fine-tuned in a supervised setting, achieving a classification accuracy of 85.86%. Grad-CAM is used to generate visual explanations, highlighting critical regions influencing the model's decisions. This interpretable, data-efficient approach outperforms conventional CNN methods, offering improved diagnostic accuracy and enhanced trust in automated pathology.

## Background:

Colorectal cancer (CRC) is the third most common type of cancer worldwide, following lung and prostate cancer in males and breast and lung cancer in females, developed from the parts of the intestine, mainly, the regions involved are the colon and rectum [1]. In 2020, more than 1.9 million new cases of colorectal cancer and more than 9,30,000 deaths due to colorectal cancer were estimated to have occurred worldwide and by 2040, the burden of colorectal cancer will increase to 3.2 million new cases per year (an increase of 63%) and 1.6 million deaths per year (an increase of 73%) [2]. Targeted therapies that target important pro-oncogenic signaling pathways have been developed as a result of a growing understanding of CRC biology, but only a small percentage of patients respond well to these therapies [3]. With the advancements in Deep learning (DL) methods and its application in healthcare sector, digital pathology is not rescued from this and has emerged as a key tool in the identification, diagnosis and prognosis of tumours [4-5]. A number of studies utilizing artificial intelligence (AI) techniques for digital histopathology have tremendously increased in recent years [6, 7-8]. Among various imaging modalities, histopathological image analysis stands out as a critical tool due to its ability to provide detailed insights into cellular structures, essential for the accurate diagnosis of cancers such as colorectal cancer [3]. However, traditional methods of histopathological image interpretation have predominantly relied on manual examination by histopathologists; therefore, there is an urgent need for automated, high-performance diagnostic systems to assist clinicians in making faster and more accurate decisions [9]. Deep learning models, particularly Convolutional Neural Networks (CNNs), have shown state-of-the-art performance in image classification tasks by learning hierarchical feature representations from raw data [10]. These models can automatically extract intricate features from histopathological images, which greatly minimizes the need for manual feature engineering and CNNs have been successfully used in a wide range of medical imaging applications, from tumor detection in radiology images to organ segmentation in MRI scans [11]. Their robust generalization capabilities across different datasets, such as histopathological images, have been extensively shown in recent research [12-13]. Several studies have been conducted on histopathological colorectal images, for diagnosis and classification of adenoma, or polyps [14- 15]. Dif *et al.* in a paper proposed a deep learning-based CNN approach for histopathology colorectal classification [16]. Wei *et al.* utilized the simple CNN based deep learning architecture for the colorectal cancer polyp classification 17]. Gupta *et al.* [18] also proposed the CNN based model IR-v2 Type 5 on the whole slide images of histopathology colon tissue that are classified into normal and abnormal patches. The paper proposed MA_ColonNet, a CNN based model that distinguishes Colon Adenocarcinoma and Colon Benign Tissue of Colon Histopathological Images [19]. Despite the enormous success of DL techniques in imaging tasks, deep learning models typically require large amounts of labeled datasets for training and the scarcity of labeled data in medical domain, necessitates the development of self-supervised algorithms, where models are trained without labeled data [20]. One of these promising approaches is contrastive learning where frameworks such as SimCLR and MoCo [21], focuses on learning representations by comparing positive (similar) and negative (dissimilar) pairs of images by reducing the contrastive loss function that enable them to learn discriminative features for crucial tasks, such as classification where the availability of annotated medical data is limited [22]. This study utilizes self-supervised contrastive learning (SSCL) to enhance the learning of robust and discriminative features from unlabeled colorectal cancer histopathological images applying CNN based ResNet50 architecture as an encoder backbone for fine-tuning and later projection head and classification head are added on the base encoder to classify the colorectal cancer histopathological images distinguished into hyperplastic polyp and sessile serrated adenoma [23]. To improve the interpretability and clinical relevance of the model, we integrate Grad-CAM, a visualization technique that generates heatmaps to highlight the regions of the image that most influence the model's decision with the bounding to detect the highlighted regions [24]. Therefore, it is of interest to report that by combining self-supervised contrastive learning, advanced CNN architectures, and Grad-CAM for interpretability, our framework creates a highly accurate and interpretable model for colorectal cancer classification that not only aims to enhance classification performance but also ensures the model's transparency and trustworthiness, facilitating its potential integration into clinical workflows to assist pathologists in making accurate and timely diagnosis, ultimately improving patient outcomes and advancing computational pathology practices.

## Materials and Methods:

This section describes the steps starting from data collection to model building, training and prediction on the colorectal cancer histopathological images for classification.

## Data collection and distribution:

Dataset used in the present study is taken from the MHIST - a publicly available dataset [25]. Dataset has two classes - namely hyperplastic polyp (HP) and sessile serrated adenoma (SSA). This dataset comprises 3,152 hematoxylin and eosin (H & E) - stained Formalin Fixed Paraffin-Embedded (FFPE) fixed-size images of colorectal polyps of which 2162 belongs to HP class while 990 belong to SSA class. Since, there is an imbalance in the dataset; I used 990 images from the HP category to balance the dataset with the SSA category. HPs are typically benign with elongated polyps while SSAs are precancerous lesions with broad-based crypts. The dataset (total images - 1980) is divided into training (1782) and testing (198) sets in a ratio of 90:10. Figure 1 (see PDF) presents the bar and pie charts for the data distribution for both the classes and data splitting into training and testing sets.

## Data pre-processing:

Data pre-processing is a crucial step for training any deep learning model. In a self-supervised contrastive learning approach, data augmentation plays an important role. Various augmentations were applied to the training data such as random crop, random horizontal flip and random vertical flip, gaussian blur, random affine, and color jittering. Normalization is applied so that the pixel intensities lie in range 0-1. Each image generates two views after the augmentation technique for creating the positive and negative pairs as shown in Figure 2 (see PDF), which are necessary for training the SSCL model. Figure 2 (see PDF) represents the original images from each class before pre-processing and after applying the augmentation techniques.

## Self - supervised model training:

Self-supervised contrastive learning framework relies on unlabeled data for learning the complex patterns in the images that discriminate between the positive pairs by pulling them together from the dissimilar pairs by pushing them apart. In this contrastive learning framework, I have implemented ResNet50 architecture as an encoder backbone. Last few layers from the architecture were removed and a linear custom projection head was built on top of that for learning the features from the positive and negative pairs. That forms the SSL pre-training part in which unlabeled data was fed as an input for training. On top of that, a classification head was built. After training on the unlabeled dataset, finetuning was done with labeled data and finally, binary classification was performed on the test data for categorizing the histopathological images into HP and SSA classes, respectively. Contrastive loss plays a major role. I have used the NT-Xent [26] loss function for representative learning. Temperature is an important parameter in this loss function which has been set to a value of 0.7. All the work has been done using python [27] and pytorch [28] libraries on Nvidia RTX 1070 GPU machine. Figure 3 (see PDF) illustrates the methodology employed in the research.

## Explainable AI (GradCAM) technique:

Hyperparameters are the parameters that are explicitly defined before the training process. They are important for model training. Table 1 (see PDF) presents the hyperparameters employed during the training process.

## Evaluation and prediction:

After successful training of the model, prediction was done on the test dataset. The performance of the trained model was evaluated by calculating the performance metrics such as accuracy, precision, recall and f1-score. All these metrics are calculated as given by equations shown.

Accuracy = (TP+TN)/(TP+TN+FP+FN)

Precision = TP/(TP+FP)

Recall = TP/(TP+FN)

F1-score = (2*Precision*Recall)/(Precision+Recall)

Where, TP = True Positive, FP = False Positive, TN = True Negative, and FN = False Negative.

Besides measuring the above metrics, I have plotted the confusion matrix representing the ground truth and predicted labels in each class. To understand the decision taken by the model, interpretable Gradient-weighted Class Activated Mapping (GradCAM) technique is applied and identify which specific regions of the histopathological images have been considered while making decision.

## Results:

To assess the model's performance on colorectal cancer histopathological images, the evaluation metrics are calculated and have been tabulated in Table 2 (see PDF). Accuracy achieved is 85.86%, while the precision and recall for both the classes i.e., HP and SSA are 86.61% and 84.85%, and 85.15% and 86.87, respectively. These outcomes suggest that the model is trained well enough to capture the complex patterns in the cancerous images, classifying the images with high accuracy. Confusion matrix has been represented in Figure 4 (see PDF), providing useful insights into the model's performance suggesting that the model is able to identify and classify the images well. Figure 5 (see PDF) represents the GradCAM visualization maps of some image samples in which specific regions are identified and highlighted. Bounding boxes have been drawn for better visibility that are most influential contributing to the model's prediction, helping in the interpretability of the framework. Sub Figure 5(a) (see PDF) shows the original hyperplastic polyp image, with the heatmap and bounding box drawn on the original image overlapping the heatmap. Similarly, Figure 5(b) (see PDF) presents the sessile serrated adenoma histopathology image.

Subfigure (a) Left: Original hyperplastic polyp image.

Middle: GradCAM heatmap highlighting the most influential region contributing to the model's prediction.

Right: Model correctly predicted the image as HP with the bounding box overlaid on the original image.

Subfigure (b) Left: Original Sessile Serrated Adenoma image.

Middle: GradCAM heatmap for model's prediction.

Right: SSCL Model predicted as SSA

## Discussion:

This study demonstrates the promising potential of self-supervised contrastive learning (SSCL) in classifying colorectal cancer histopathology images, addressing one of the major challenges in medical imaging: the scarcity of large annotated datasets. Unlike traditional supervised deep learning, which requires extensive labeled data, SSCL enables the model to learn rich and discriminative features from unlabeled histopathological images. These images, though plentiful, often remain underutilized in clinical diagnosis due to the annotation bottleneck. The proposed method employs a ResNet50 encoder backbone, pre-trained with contrastive learning, and subsequently fine-tuned in a supervised manner to differentiate between two colorectal lesion types: hyperplastic polyp (HP) and sessile serrated adenoma (SSA). The model achieved a classification accuracy of 85.86%, along with balanced precision and recall for both classes, indicating that it effectively captured subtle morphological differences between tissue structures. This performance rivals previous studies relying mainly on fully supervised methods and large annotated datasets. Contrastive learning works by forming positive and negative image pairs through augmentation techniques, teaching the model to become invariant to typical histological variations such as orientation, scale, and staining differences. These natural variations are common due to sample preparation and scanning inconsistencies in histopathology. The study applied augmentations like random cropping, flipping, color jitter, and Gaussian blur to enrich the feature space and guide the model to focus on biologically relevant structures instead of superficial pixel differences. The use of the NT-Xent loss function with a temperature parameter of 0.7 further refined the embedding space, encouraging closer clustering of similar images and better separation of dissimilar ones, thus enhancing class discrimination.

A critical contribution of this work is the integration of interpretability through Gradient-weighted Class Activation Mapping (GradCAM). This technique addresses a key challenge in clinical AI deployment: transparency. GradCAM visualizations produced heatmaps highlighting regions that pathologists consider diagnostic, such as glandular architectures and cell morphology, boosting confidence in the model's predictions. Beyond validation, these interpretability maps can serve as diagnostic tools, potentially revealing subtle morphological features significant for early detection and differential diagnosis. This is especially relevant in distinguishing benign HP from SSA, a neoplastic precursor to colorectal cancer, which has important implications for patient management. The model's performance was further validated through confusion matrices and balanced F1-scores, confirming its robustness in handling class imbalance. The researchers curated the dataset by downsampling the dominant HP class to match SSA quantities, promoting fair learning and reducing bias. However, they acknowledge that future work could explore more advanced imbalance management techniques, such as synthetic minority oversampling or focal loss, to improve performance on naturally imbalanced datasets without losing valuable information. While the results are encouraging, the study highlights several avenues for future improvement and expansion. The current focus on binary classification between HP and SSA represents a simplified clinical scenario. Extending the system to multiclass classification, including other adenoma types, carcinoma, and normal tissue, would enhance clinical applicability. Fortunately, the scalability of self-supervised pretraining allows leveraging vast quantities of unlabeled whole-slide images (WSIs) to build more generalizable feature extractors. Additionally, the use of ResNet50 as the backbone could be updated in future work. More advanced architectures, such as vision transformers or domain-adapted convolutional neural networks optimized for histopathology, may deliver superior performance in capturing both global context and fine-grained patterns. Another key direction is multimodal learning, integrating clinical metadata like patient age, gender, genetic markers, and clinical history with image data. Such integration would more closely mimic real-world diagnostic decision-making by pathologists, potentially improving accuracy and personalized diagnosis. From a practical standpoint, clinical integration demands rigorous validation across independent cohorts and different scanning platforms to ensure robustness and generalizability. Prospective clinical trials will be essential to evaluate the impact of the AI system on diagnostic accuracy, workflow efficiency, and patient outcomes. Engaging pathologists in refining the user interface, especially through interactive visualization tools for GradCAM heatmaps, will be crucial to facilitate smooth adoption and clinical trust. In conclusion, this research underscores the significant advantages of self-supervised contrastive learning for colorectal cancer histopathology classification. By addressing the annotation bottleneck and providing interpretable decision-making via GradCAM, the approach achieves competitive classification performance while enhancing clinical transparency and trust. The synergy of self-supervised learning and interpretability marks an important milestone toward AI-assisted precision diagnostics. With continued advancements in model architectures, dataset diversity, and multimodal fusion, SSCL-based frameworks are well-positioned to revolutionize colorectal cancer pathology, ultimately benefiting patients through earlier, more accurate diagnoses.

## Conclusion:

Early prediction of colorectal cancer significantly aids in accurate diagnosis and effective treatment planning. This study successfully applied a self-supervised contrastive learning approach using CNNs to classify histopathological images into HP and SSA, achieving an accuracy of 85% and demonstrating strong performance across various evaluation metrics. Additionally, the use of Grad-CAM for model interpretability provided valuable insights into the decision-making process, enhancing clinical confidence and supporting improved patient care.
